# Single-detector 3D optoacoustic tomography via coded spatial acoustic modulation

**DOI:** 10.1038/s44172-022-00030-7

**Published:** 2022-10-19

**Authors:** Evgeny Hahamovich, Sagi Monin, Ahiad Levi, Yoav Hazan, Amir Rosenthal

**Affiliations:** Technion—Israel Institute of Technology, Haifa, Israel

**Keywords:** Imaging and sensing, Biomedical engineering

## Abstract

Optoacoustic tomography (OAT) is a hybrid imaging modality that combines optical excitation with ultrasound detection and enables high-resolution visualization of optical contrasts at tissue depths in which light is completely diffused. Despite its promise in numerous research and clinical applications, OAT is limited by the technological immaturity of ultrasound detection systems. It suffers from limited element count, narrow field of view and lack of technology for spatial modulation of acoustic signals. Here we report single-detector OAT capable of high-fidelity imaging using an amplitude mask in planar geometry coded with cyclic patterns for structured spatial acoustic modulation. Our image reconstruction method maximises sensitivity, is compatible with planar signal detection, and uses only linear operations, thus avoiding artefacts associated with the nonlinear compressed-sensing inversion. We demonstrate our method for 3D OAT of complex objects and living tissue performed with only a single ultrasound detector, effectively coded into a 2D array with 1763 elements. Our method paves the way for a new generation of high-fidelity, low-cost OAT systems.

## Abstract (web summary)

Optoacoustic tomography (OAT) is a hybrid imaging modality that combines optical excitation with ultrasound detection. However, OAT is limited by the lack of available high resolution ultrasound detection systems. Here Hahamovich and colleagues report single-detector OAT capable of high-fidelity imaging using an amplitude mask in planar geometry coded with cyclic patterns for structured spatial acoustic modulation.

## Introduction

OAT offers label-free visualization of the optical contrast inside living tissue at depths of up to a few centimeters with high spatial resolution. In this imaging method, the object is illuminated by laser pulses that create thermal expansion within the object, leading to the generation of outward propagating acoustic waves^1^. The acoustic signals may be measured on the surface of the illuminated object using ultrasound detector arrays and used to produce a volumetric image of the optical absorption of the object using tomographic inversion algorithms^1^. Since the detection is of acoustic, rather than optical, signals, optoacoustic imaging can achieve resolutions comparable to those of medical ultrasound for imaging depths of several centimeters, going well beyond the capabilities of purely optical methods, limited by the diffusion of light^2^. When the illumination is performed at several wavelengths, in a technique known as multi-spectral optoacoustic tomography (MSOT), different tissue constituents may be identified based on their optical absorption spectrum^3^.

MSOT have been demonstrated in numerous preclinical application^4–7^, including brain imaging^8^, hemoglobin concentration and oxygenation monitoring in blood^9,10^, dermatology^11^, and dental imaging^12^. In addition to preclinical demonstrations, MSOT has shown potential as a diagnostic tool in numerous clinical applications^13^, e.g., malignant lesions identification in breast cancer^14^, endocrinology by noninvasive diagnostics and characterization of thyroid disorders^15^ and microvascular imaging^16^, improved prostate cancer diagnostics^17^, detailed vascular network mapping^18^, noninvasive prostate cancer diagnostics^17^, and gynecologic imaging^19^.

**Fig. 1:**
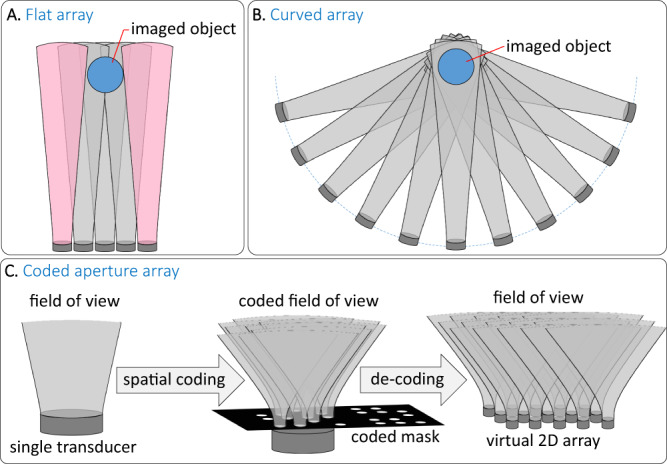
Detection geometries for OAT. **A** Planar geometry piezoelectric array, which suffers from the low acceptance angle of the piezoelectric elements. **B** Curved array. From basic geometrical considerations, curved array must be larger than planar array to assure that the object is within the detection angle of all the detectors. **C** The proposed method with a coded acoustic mask used to form a virtual detection array on a flat surface with a large acceptance angle.

To achieve high-fidelity imaging, both single-wavelength OAT and MSOT require multi-element detection arrays with sensitive detection elements and a wide angular acceptance^20^. Conventionally, piezoelectric detectors are used because of their high sensitivity and the commercial availability of multi-element arrays. However, the limited acceptance angle of piezoelectric detectors leads to a significant loss in lateral resolution in planar configurations since the optoacoustic signals are detected by only a small portion of the available detectors (Fig. 1A). Achieving high lateral resolution with piezoelectric transducers generally requires the use of curved detection surfaces (Fig. 1B) in which the signals from the imaged object can be detected by all the elements of the array. However, as illustrated in Fig. 1B, the use of a curved geometry requires detection surfaces that are much larger than the desired field of view, leading to cumbersome devices which may not be compatible with the restrictions of the clinical practice. Moreover, producing dense arrays is more challenging on a curved geometry, and the number of elements demonstrated so far has been limited to merely 512^21,22^, whereas high-fidelity 3D OAT requires 1000 s of detectors. Using denser arrays would also require more complex data-acquisition systems to digitize the acoustic signals and would come at the cost of reduced sensitivity since the detected acoustic signals generally scale with the detector area^23^. An additional drawback of curved arrays is their lower achievable frequency compared to flat arrays, which limits the imaging resolution. While flat ultrasound arrays are commercially available with central frequencies up to 50 MHz^24^, curved arrays commonly operate at frequencies below 10 MHz^25,26^.

It has been previously shown that the deficiencies of piezoelectric technology may be alleviated algorithmically by exploiting the unique characteristics of the imaged object in nonlinear-image-reconstruction algorithms: Sparsity-based inversion algorithms enabled reduction in the density of the piezoelectric elements, below the theoretical limit subscribed by the Nyquist–Shannon sampling theorem and deep-learning algorithms improved imaging performance in the limited-view scenarios^27–30^. Extensions of these algorithmic concepts have also led to the addition of acoustic elements to the imaging setup in order to randomize the acoustic data, enabling optimal use of compressed-sensing algorithms^31^. In ref. ^32^, Dean-Ben et al. achieved randomization using acoustic scatterers, demonstrating real-time 2D OAT with 16 elements, and in ref. ^33^ Kruizinga et al. achieved signal randomization by a randomly disordered acoustic phase mask, demonstrating 3D ultrasound imaging from a sequence of measurements performed during mask rotation. Another hardware-based alternative for ultrasound spatial coding suggested the use of air micro-bubbles controlled by an electronic chip serving as local ultrasound blockers^34^. This approach suffered from very slow pattern formation times of 12 s per pattern, impractical for many imaging applications, and required complex electronic solutions for controlling the positioning of the formed bubbles that currently suffer from parallelization challenges.

While the use of nonlinear inversion, and compressed sensing, in particular, has enabled a drastic reduction in the number of used detection elements, it involves several disadvantages that limit its use for preclinical and clinical OAT. First, the image quality demonstrated in refs. ^32^ and ^33^ was lower than the one conventionally achieved by full array systems. Second, nonlinear inversion reduces the accuracy of the spectral analysis performed by MSOT and can eliminate subtle changes in the imaged volume during functional imaging experiments^35^ that could otherwise be detected in linear inversion. Third, in the case of 3D imaging, the run time of nonlinear algorithms can be prohibitive, limiting their use in clinical applications. Finally, methods based on randomization require initial system calibration to achieve effective coding, and data-based algorithms, such as deep learning, require large datasets for training, which are not always available.

In this work, we developed a scheme for single-detector OAT capable of high-fidelity imaging in a planar geometry without relying on nonlinear inversion algorithms or on regularization. Our scheme uses a mask of acoustic apertures that are produced according to structured, rather than random, codes to produce a virtual 2D array of acoustic detectors (Fig. 1C). The apertures serve two functions: First, they perform binary spatial modulation on the amplitude of the acoustic field that leads to a multiplexed acoustic measurement, i.e., a measurement that is a weighted sum of the response of each individual virtual detector^36^. Second, they increase the angular acceptance of the detector, thus facilitating more isotropic image reconstructions^1^. The increase in angular acceptance is a result of acoustic diffraction, which leads to semi-isotropic angular sensitivities for wavelength-sized apertures^37^. To generate the virtual detector array, the coded mask is scanned in front of the detector, where for each position of the mask, the acoustic waveform is multiplied by a different binary pattern and spatially integrated by the single detector, recording an acoustic signal for each mask position. Using algebraic inversion, the signals measured for each mask position are used to reconstruct the signals per element in the virtual 2D detector array. Since the inversion is linear, it is compatible with spectral analysis performed in MSOT and preserves minute temporal changes in the optoacoustic image, which are essential in functional imaging.

Coded masks have been previously used in optics to perform rapid single-pixel imaging using 1D mask scanning. Since the optical wavelength is typically on the scale of 1 µm, a virtual 2D array could be coded on a mask with an acceptable size. In the case of ultrasound, the much larger wavelengths make such a 1D-scan scheme impractical, with typical mask lengths of several meters. In this work, the use of a denser arrangement of the code on the mask, designed for 2D scanning, led to a manageable scan of 40 mm in each direction.

The limited space forced by the water tank combined with large ultrasound wavelengths and high order codes prevents us from translating the coding base into a linear cyclic mask^38^ that would require an available scanning length of a few meters. In this work, we overcome this issue by translating the coding matrix into a 2D scanning formation, significantly reducing the motion span of the coding mask to merely 40 mm in each direction. Additionally, 2D scanning has better robustness to alignment errors of the systems. For example, a small error with the mounting angle of the scanning stages might cause noticeable distance mismatches between the mask and the transducer using the linear scan.

The ability to perform stable linear inversion, without the need for regularization, heavily depends on the mathematical arrangement of the apertures. Specifically, the shifted patterns need to form a full basis that corresponds to a well-conditioned transformation matrix. In this work, we use cyclic Hadamard codes, which achieve the maximum signal-to-noise (ratio) SNR possible for binary codes and possess an exact closed-form inversion formula. In comparison to a single aperture, the SNR gain obtained for a virtual array with *N* elements is $\sqrt{N}/2$ ^36^. In our implementation, an array with 1763 virtual elements was produced, corresponding to a theoretical SNR gain of ~21. Using the formed virtual detection array, 3D OAT was performed on a complex phantom and a mouse paw in vivo with axial and lateral resolutions of 320 and 480 μm.

## Results

### Signal multiplexing via coded apertures

**Fig. 2:**
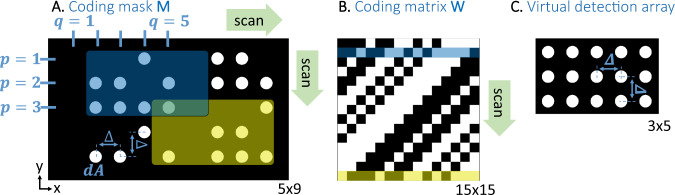
Coding method illustration for a 2D array with 15 elements. **A** is the coding mask that is physically scanned in front of the detector in a 2D formation where the mask area appears in black and the apertures in white. p and q indicate the scanning step in the vertical and horizontal axes and are equivalent to the size of the chosen coding matrix, Δ is the shift step the grid moves in each direction and *dA* is the size of a single coding element. **B** is the equivalent coding matrix, where the used elements are in white and the blocked/masked elements in black. **C** is the resulting detection array configuration with 3 × 5 2D formation, formed after de-multiplexing a full set of 15 multiplexed signals. The green scan lines represent the 2D scan formation of the coding mask and the fluent propagation of the projected codes through the coding matrix. The blue and yellow colors represent two different placements of the coded mask and the compatible base vectors on the coding matrix.

To detect the pressure of ultrasound waves at multiple positions over a plane, $p(x,y,t)$, an acoustic mask with coded transmission apertures is positioned in the desired detection plane. The apertures have an identical shape with an area of ${dA}$ and are coded on a Cartesian grid with a step size of Δ, i.e., the apertures may appear only on the grid points (Fig. 2A). The output of the mask represents the product of the ultrasound wave with a binary pattern ${{{{{\rm{M}}}}}}(x,y)$, which is equal to ‘1’ at the positions of the apertures, and to ‘0’ elsewhere. An ultrasound detector is positioned at the output of the mask, resulting in a signal that is described by spatial integration of the transmitted wave over the mask area, $A\gg {dA}$. The mask is translated on both axes in discrete steps of Δ, and the acoustic measurement is repeated for each mask position, resulting in a set of measured signals (Fig. 2B):

1 $${u}_{q,p}\left(t\right)={{{\int }}{{\int }}}_{A}p\left(x,y,t\right){{{{{\bf{M}}}}}}\left(x-q\Delta ,y-p\Delta \right){da},$$

where $q=1,\ldots Q$ and $p=1,\ldots P$ are integers representing the spatial shift of the mask in the *x-* and *y-*axes, respectively. Taking the average pressure $p(x,y,t)$ over the aperture area ${dA}$ Eq. (1) can be written as

2 $${u}_{q,p}\left(t\right)={dA}\mathop{\sum }\limits_{i=1,j=1}^{I,J}p\left(i\Delta ,j\Delta ,t\right){{{{{{\bf{M}}}}}}}_{i+q,j+p},$$

where *I* and *J* are the maximum indices covered by the scan and ${{{{{{\bf{M}}}}}}}_{i,j}$ is 1 at the coordinates of the apertures and 0 elsewhere.

Equation (2) represents a multiplexed measurement in which the detected acoustic signals are a linear combination of the acoustic pressure values at different spatial positions. The goal of the measurements is to ultimately recover the values of $p(x,y,t)$ on the detection grid, representing a virtual detector array, as illustrated in Fig. 2C. While there are numerous ways of implementing a multiplexed measurement, e.g., using acoustic scatterers or phase masks, the advantage of the proposed amplitude masks is that the weights in Eq. (2) are a direct discrete representation of the patterns produced on the mask and do not require preliminary calibration measurements in order to be determined. Accordingly, any desired weights in Eq. (2) may be readily produced experimentally, enabling the use of structured codes with desired properties. In contrast, when the multiplexing is mediated by a complex physical phenomenon^32^, only random weights may be readily implemented.

In order to invert Eq. (2), it should first be reformulated in a matrix form, in which both ${u}_{q,p}\left(t\right)$ and $p\left(i\Delta ,j\Delta ,t\right)$ for a given time instance $t={t}_{k}$ are arranged into 1D column vectors ${{{{{{\bf{u}}}}}}}_{{{{{{\bf{k}}}}}}}$ and ${{{{{{\bf{p}}}}}}}_{{{{{{\bf{k}}}}}}}$ with lengths of ${QP}$ and ${IJ}$, respectively. Using the vectors ${{{{{{\bf{u}}}}}}}_{{{{{{\bf{k}}}}}}}$ and ${{{{{{\bf{p}}}}}}}_{{{{{{\bf{k}}}}}}}$, Eq. (2) takes the form of

3 $${{{{{{\bf{u}}}}}}}_{{{{{{\bf{k}}}}}}}={{{{{\bf{W}}}}}}{{{{{{\bf{p}}}}}}}_{{{{{{\bf{k}}}}}}},$$

where **W** is an ${QP}\times {IJ}$ coding matrix whose elements represent a rearrangement of **M** from Eq. (2). Accordingly, to recover $p(x,y,t)$ over the mask grid, Eq. (3) needs to be inverted for every value of *k* of interest. It is important to note that **W** is not an arbitrary ${QP}\times {IJ}$ binary matrix but is rather generated from duplications of the $(I+Q-1)(J+P-1)$ distinct entries of **M** in Eq. (2). In addition, a stable inversion of **W** in the presence of noisy data is desired for regularization-free inversion.

To address the two aforementioned requirements on **W**, we use a cyclic S-matrix—a square matrix in which each row is equal to the circular shift of its preceding row (Fig. 2B)^39^. The cyclic S-matrix is known to achieve the optimal SNR for a binary matrix of 1’s and 0’s and may be mapped to the 2D scanning pattern of Eq. (2). In this work, we use the Twin-prime algorithm to produce a binary code with a length of ${PQ}$, where $P=Q-2$ and both *P* and *Q* are prime numbers. The binary code is used to create the first row of **W** and is cyclically shifted to produce a square matrix with the size of ${PQ}$, corresponding to a mask with $I=P$ and $J=Q$. For these parameters, and under the assumption of additive noise, which is the noise model in acoustic measurements, the inversion of Eq. (3) with an S-matrix achieves an SNR gain of approximately $\sqrt{N}/2$ compared to a direct measurement in which **W** is the identity matrix^36^, i.e., a measurement in a which a single aperture is scanned in front of the detector, where $N={PQ}$ is the number of multiplexed locations on the detection grid (Fig. 2A). Details of the Twin-prime algorithm and the mapping procedure between the coding matrix **W** and the code imprinted in the acoustic mask **M** are given in Supplementary Notes 1 and 2, respectively, and a detailed scanning example for a low-order 2D coding example shown in Supplementary Fig. 1. Coded mask production details and the used hardware and configuration details are described in the methods section.

### Analysis

A single multiplexing operation, described by Eq. (3), includes the measurements of a single vector with a length of *N*, describing the acoustic measurement for *N* mask positions for a given time instance. Better coverage of the detection surface may be achieved by finer resolution scanning with steps of $\Delta /\alpha$ and $\Delta /\beta$ over the vertical and the horizontal axes, increasing the measurements set to *α* × *β* interlaced matrices. Assuming that the signals are acquired for ${N}_{t}$ time instances, the complete acoustic measurement may be stored in a single matrix, denoted by **Y**, with a size of $N\times \left(\alpha \beta {N}_{t}\right)$. To de-multiplex the signals stored in **Y**, we first calculate the inverse of the multiplexing matrix **W** (Eq. (3)) using a known analytical construction, described in Supplementary Note 1. Since **W** is well-conditioned, no regularization is required, and the de-multiplexed signals are obtained via a simple matrix multiplication:

4 $${{{{{\bf{P}}}}}}={{{{{{\bf{W}}}}}}}^{-1}{{{{{\bf{Y}}}}}}$$

where **P** is a $N\times \left(\alpha \beta {N}_{t}\right)$ matrix in which each row contains the acoustic signals over the virtual detector for a specific time instance over a 2D grid. In the next step, we rearrange the elements of **P**, in which $\alpha \beta N$ acoustic waveforms are arranged in $\alpha \beta$ interleaved grids shifted by a sub-Δ spatial offsets of $\left(\Delta /\alpha ,\Delta /\beta \right)$, each having *N* positions, in a new matrix **P**’, with the dimensions $\alpha P\times \beta Q\times {N}_{t}$, representing the temporal signals over a single 2D grid with $\alpha P\times \beta Q$ pixels.

**Fig. 3:**
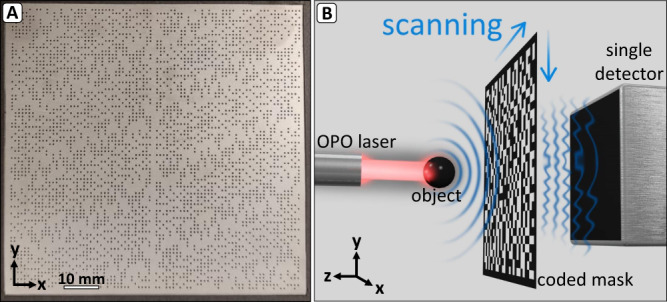
Method illustration. **A** is the photograph of our coded mask. **B** is the measurement system setup in which the acoustic signals are generated by excitation of the sample with a pulsed laser and detected by a single, large-aperture single ultrasound detector masked by a coded grid, scanned in the *x-y* plane.

The signals passing through the coded mask propagate in free space until reaching the single detector, and while the detector captures the signals that correspond to spatial modulation on the plane of the mask, its waveform includes an additional delay from the mask to the detector. To temporally shift the signals to the proper detection plane, the time axis of the acoustic signals is corrected by subtracting the propagation time from the mask to the detector:

5 $${t}_{{{{{{\rm{grid}}}}}}}={t}_{{{{{{\rm{sampled}}}}}}}-d/c$$

where ${t}_{{{{{{\rm{sampled}}}}}}}$ is the time vector of the measured signal on the detector surface, ${t}_{{{{{{\rm{grid}}}}}}}$ is the shifted time vector of the signals at the surface of the mask, and c is the speed of sound in water. We note that since the mask plane is parallel to the detector plane, the parameter *d*, which represents the distance between these two planes (Fig. 3B), is constant for a given measurement. The resulting signals correspond to the waveforms measured by the virtual array on the surface of the mask and may be used to form the OAT image using tomographic inversion^1^. In this work, we use the universal back-projection algorithm^40^ for image formation and 3D PHOVIS^41^ for 3D visualization of the produced reconstructions.

### System characterization measurements

Two measurement sets were performed to characterize the proposed detection setup. First, the noise-equivalent pressure of the coded array was measured by a hydrophone (setup details are found in the methods section). Achieving a maximum peak-to-peak pressure detected by the calibrated hydrophone of 0.09 MPa, while the maximum SNR of the virtual detector was 53 dB at the same position in its function frequency band 3.1–6.5 MHz, indicating a noise-equivalent pressure of 200 Pa for a single virtual element of the proposed coded detector.

**Fig. 4:**
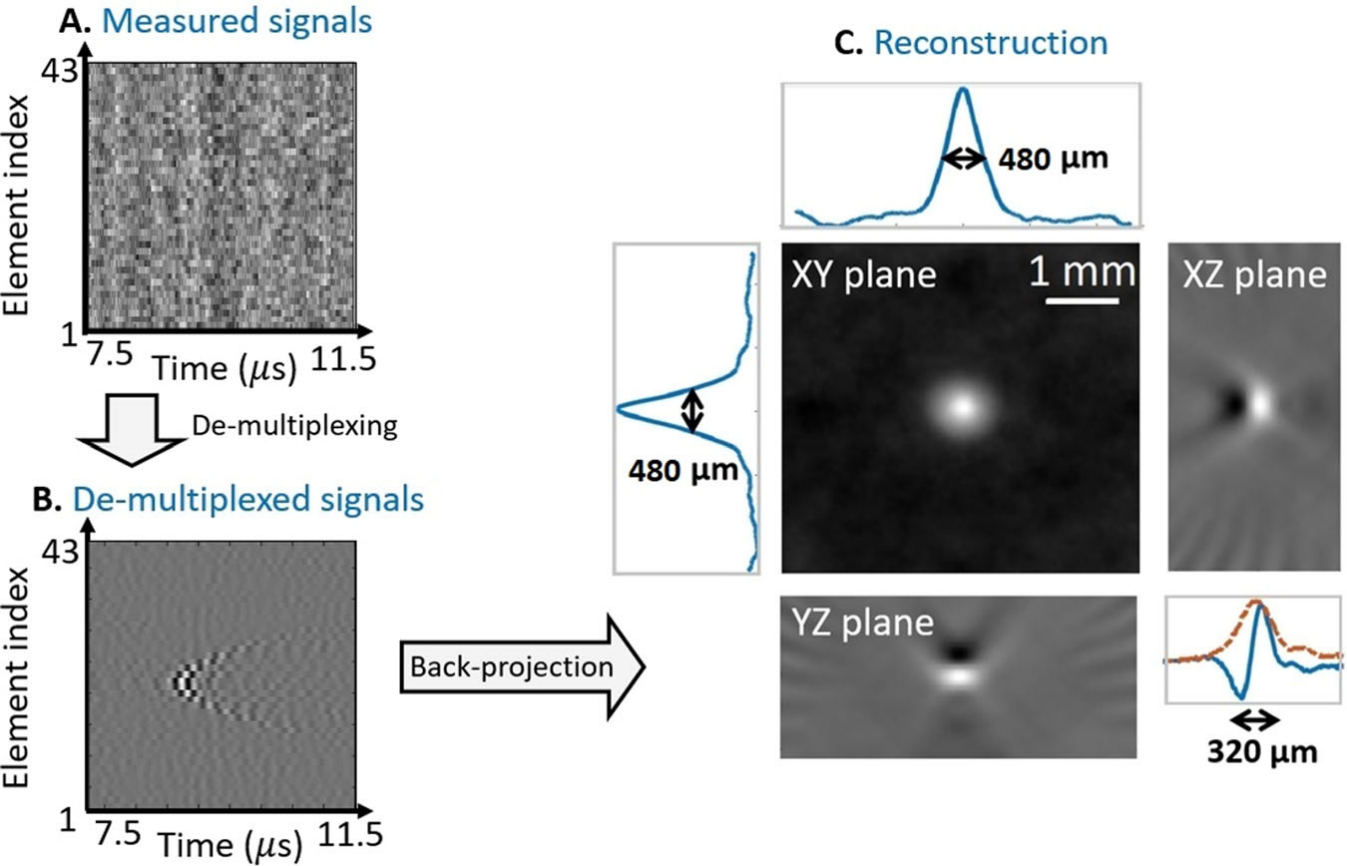
Point source imaging results. **A** is the measured, multiplexed signals, showing a subset of 43 measured signals over a central vertical line of the single-element transducer is shown. **B** is the acoustic signals after de-multiplexing on the same central line of the transducer and **C** is the reconstructed, maximum amplitude projection of the optical density over the distance from the detector (*z*). The amplitudes are in arbitrary units.

Second, the resolution of the coded detector was measured by OAT imaging of a point source (described in detail in the methods section). Figure 4A shows the raw acoustic signals measured over a single vertical scan with 43 sampling positions. Since the raw signals correspond to the multiplexed measurement in which the weights possess a complex structure (Eq. (3)), no visible features can be easily identified in the image. Figure 4B shows the de-multiplexed signals over the same vertical scan of the virtual detection, obtained by applying Eq. (4). A clear hyperbolic structure is visible in the image, corresponding to the classical sinogram of a point source under linear scanning^42^. Figure 4C shows the central slices of the 3D OAT reconstruction over the three principal axes, revealing axial and lateral resolutions of 320 and 480 μm, defined by the full-width-at-half-maximum of the reconstruction. In the *z-*direction, the reconstruction exhibited negative values due to the lack of low frequencies in the transducer response. Accordingly, the reconstruction width in the *z*-direction was performed on the envelope of the reconstruction (dashed curve).

### Complex object OAT

**Fig. 5:**
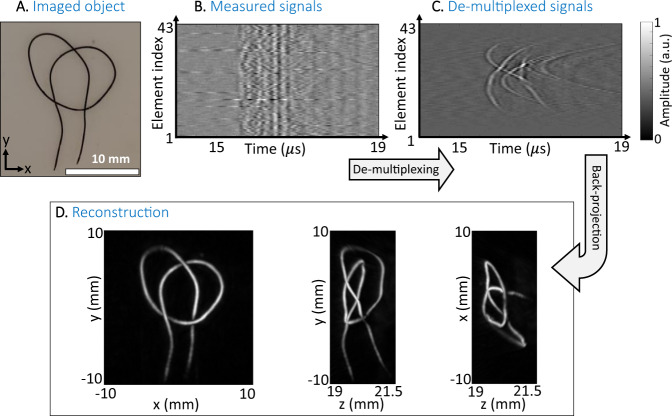
Suture imaging results. **A** is a photograph of the imaged suture. **B** is the measured signals from a subset of a single vertical line at the center of the coded detector, **C** is the de-multiplexed signals, and **D** is the reconstructed, maximum amplitude projections of the optical density, observed from three different sides. The amplitudes are in arbitrary units.

To demonstrate the 3D imaging capabilities of our method, OAT was performed on a complex 3D structure produced by black suture. The suture, shown in Fig. 5A, was made from a 200 μm thick copper wire coated with Indian ink and was embedded in a two-layer agar phantom. The layer facing the laser was transparent, enabling optimal delivery of the illumination to the suture, whereas 5% lipids were added to the agar facing the mask, making it optically opaque. The suture was placed between the two layers. The agar phantom was illuminated from its transparent side by pulses with a wavelength of 720 nm and energy of 34 mJ, while the opaque-agar layer served to homogenize the illumination delivered to the suture and reduce unwanted illumination of the acoustic mask. The distance between the suture and the mask was 23 mm, and the distance between the mask and the single detector was 3 mm. The mask was scanned with discrete steps of $\Delta /2=500$ ${{{{{\rm{\mu }}}}}}{{{{{\rm{m}}}}}}$, leading to four interlaced multiplexed sample sets, two per axis, which were combined into a single virtual detector array with an element pitch of $500\,\mu {{{{{\mathrm{m}}}}}}$ after de-multiplexing.

The imaging results are summarized in Fig. 5. Figure 5B and C shows a subset of the measured raw signals and de-multiplexed signals, respectively at the central vertical line on the transducer, whereas Fig. 5D shows the MAPs of the resulting 3D optoacoustic reconstructions calculated over the axial (*z*-axis) and lateral (*x-* and *y*-axes) directions. The complex structure of the suture is clearly visible in all three MAP images, demonstrating the capability of the system for high-fidelity imaging. A video of the suture rotating about the *x*-axis is found in Supplementary Movie 1.

### In vivo OAT

**Fig. 6:**
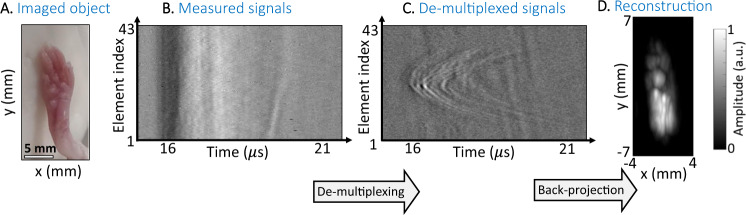
Mouse leg imaging results in vivo. **A** is a photograph of the leg. **B** is a subset over a vertical line of the measured signals, **C** is the de-multiplexed signals, and **D** is the MAP of the reconstructed optical density as a function of depth (*z*). The amplitudes are in arbitrary units.

To demonstrate the capability of our system for in vivo imaging, we imaged the leg of a mouse (Fig. 6A). The mouse was anesthetized using isoflurane and placed in a water bath heated to 35 °C to maintain its body temperature. The mouse leg was illuminated by a laser with 532 nm wavelength and pulse power of 50 mJ. The light was transmitted through a single optical fiber through a diffuser (DG10-220-MD-Ø1, Thorlabs) connected directly to the end of the fiber. The distance between the leg and the mask was 25 mm and the distance between the mask and the single detector was 5 mm. The laser operated at a repetition rate of 100 Hz, where for each laser pulse, the acoustic waveform at the output of the mask was sampled once. The acoustic mask was continuously scanned in the *x*-axis at a speed of 10 mm/s, corresponding to a discrete step size of $\Delta /10=100\,{{{{{\rm{\mu }}}}}}{{{{{\rm{m}}}}}}$ between the acoustic measurements, and scanned in discrete steps of $\Delta /2=500\,\mu {{{{{\mathrm{m}}}}}}$ in the *y*-axis. The scanning stage, laser, and sampling system were synchronized to enable accurate registration of the mask position for each measured acoustic signal. To avoid nonuniform sampling due to mask acceleration, the scan path in the *x* direction included additional acceleration and deceleration paths. Accordingly, the required acoustic measurement was performed only for the path with constant speed, whereas the signal obtained for the additional acceleration and deceleration paths was discarded. The full reconstructed set of $82\;\times\;430$ acoustic signals was used in the back-projection algorithm^40^ to form the optoacoustic image.

The measurement results are summarized in Fig. 6: A subset of the captured signals and de-multiplexed signals over the central vertical scan line is shown in Fig. 6B and C, respectively, revealing clear parabolic patterns in the de-multiplexed signals. The reconstructed optical absorption MAP is shown in Fig. 6C, and a video showing the 3D reconstruction is found in Supplementary Movie 2. We note that due to the high optical attenuation at 532 nm, most optical energy is absorbed in the external layers of the leg tissue, leading to a surface-weighted image in which the superficial structures of the leg possess a much stronger contrast than deep structures.

## Discussion

In conclusion, we developed a method for spatially coding a single, large-aperture ultrasonic detector by a coded acoustic mask, effectively transforming it into a multi-element 2D detection array. We experimentally demonstrated our method by performing OAT with a single detector coded into a 2D detection array with 1763 elements, each with a 500 μm diameter. The multiplexed measurement theoretically corresponds to a 21-fold sensitivity enhancement, where the measured noise-equivalent pressure was 200 Pa. Our numerical simulations reveal that this sensitivity enhancement is three times higher than the one obtained for a random binary mask (Supplementary Note 4 and Supplementary Fig. 3). The setup was successfully demonstrated experimentally for OAT of a complex 3D knot as well as for in vivo imaging of a mouse leg.

Our system achieved resolutions of 320 and 480 μm in the axial and lateral directions, respectively. The axial resolution was comparable to the theoretical value of $0.88c/{{{{{\rm{BW}}}}}},$ which was equal to 380 µm, where BW is the measurement bandwidth^43^. In contrast, the lateral resolution was similar to the diameter of the apertures, as expected from the theoretical analysis of ref. ^44^. Namely, in the case of planar scanning, the lateral dimension in the reconstructed image is spatially convolved with the detector aperture. Thus, smaller apertures may be used to improve the lateral resolution, leading to a more isotropic reconstruction, as experimentally demonstrated in ref. ^37^. Nonetheless, the reconstructions obtained in this work are considerably more isotropic than those conventionally obtained with piezoelectric-based OAT systems, in which the resolution difference between the axes may be more than an order of magnitude^45^. The used hardware configuration captured 17,630 acoustic signals within 4 min, limited by the 10 mm/s speed of the *x*-axis translation stage. For our current laser repetition rate of 100 Hz, translation speeds up to 100 mm/s can be performed with full sampling of the projection data, leading to imaging durations of 24 seconds.

The underlying assumption of our coded detection scheme is that the ultrasound detector operates as a bucket detector that merely integrates the acoustic signals over the surface of the mask. However, in practice, since the distance between the mask and detector, *d*, is not zero, some diffraction occurs as the acoustic wave propagates from the apertures to the detector. Nonetheless, no significant effect on the optoacoustic reconstruction was observed for distances up to 5 mm (Supplementary Note 5, with results comparison of 3 different *d* options shown in Supplementary Fig. 4), which corresponds to the far field of the aperture for a frequency of 5 MHz. This result may be explained by previous analyses that have shown that the strongest contribution to the acoustic signal measured by a flat detector is from the first contact between the spherical wavefront and detector surface in which both surfaces are tangential^46^. Thus, the effect of propagation from the apertures to the detector could be modeled, to a first-order approximation, by a mere delay of $d/c$, accounting only for the propagation in the *z*-direction. While a higher value of *d* might be possible, they would increase the effect of diffracted signals from the edge of the mask (Supplementary Note 2) and are therefore undesirable.

The main challenge in our current implementation is the difficulty of illuminating the imaged objects in reflection mode due to the opaque coding mask and acoustic detector, which blocks part of the optical path. While the current imaging experiments were performed in transmission mode, where the object is illuminated from one side and the acoustic signals a measured from the other side, in many applications, a reflection-mode geometry is preferable, where both the illumination and detection are performed from the same side. Indeed, illumination challenges are common in 3D OAT, and are often solved by creating a hole in the transducer and illuminating through it^35^. Alternatively, both the mask and the acoustic detector may be manufactured from transparent materials^47,48^, enabling one to illuminate through them in a reflection-mode configuration.

Our scheme paves the way for new types of 3D OAT systems configurations that can significantly improve performance and reduce the cost of conventional schemes. Traditionally, the high cost associated with OAT has been mostly attributed to the laser, ultrasound transducers (especially in the case of curved transducers), and data-acquisition systems^49^. With the development of pulsed laser diodes and light-emitting diodes for optoacoustic imaging, whose cost is considerably lower than those of OPOs^50,51^, the cost of many OAT configurations is now mostly determined by the ultrasound detector array and corresponding sampling electronics. Thus, combining our scheme with low-cost light sources can lead to a dramatic reduction in the price of OAT systems. We note that although single-pixel OAT may be implemented also with random acoustic scatterers^32^ or phase masks^33^, the use of such approaches would not be compatible with functional imaging due to their reliance on nonlinear inversion algorithms, whose artifacts may overshadow the minute changes between subsequent optoacoustic on which the functional analysis rely.

Our scheme may be scaled to higher resolution in which no array technology is available, offering an alternative to optoacoustic microscopy, in which acoustic data acquisition is not performed tomographically, but rather using focused ultrasound detectors that are scanned in 2D^52^. Such scaling would merely require fabricating the mask with smaller apertures and using an ultrasound detector with a higher frequency, which is commonly used in optoacoustic microscopy. The advantage of coded masks over acoustic focusing is that they enable a tomographic reconstruction that can, in principle, be used to reconstruct the entire 3D volume from a 2D scan. In contrast, when acoustic focusing is used, the performance of the system is optimal only within the depth of field of the detector, which is generally smaller than 1 mm^52^. Similarly to optoacoustic microscopy systems, a high-resolution acoustic mask may be used for imaging various skin diseases^53^, potentially offering a better tradeoff between sensitivity, depth of field, and resolution.

Finally, we note that although our technique requires only a single detector to perform 3D OAT, it may be used in combination with arrays to accelerate the imaging rate. For example, a conventional 1D array with 128 elements, which would normally be used for cross-sectional OAT, could be transformed into a 2D array capable of 3D OAT by using an acoustic mask linearly scanned in 1D. In contrast, previous implementations of high-fidelity 3D OAT with ultrasound array transducers have required complex scanning schemes that reconciled between the geometry of the array and the one required for a tomographic reconstruction^54,55^, leading to complex experimental setups and long acquisition times. Such an implementation could be beneficial for small-animal imaging, enabling rapid 3D imaging with tomographic quality in a planar geometry, thus reducing the difficulty of animal handling due to the curved transducer geometry^26,56^.

## Methods

### Production of the coded acoustic mask

The acoustic mask was formed from a 300 μm thick stainless-steel plate, in which the circular apertures were created by a pico-second laser drilling system. Due to the high acoustic impedance of steel in comparison to water, it effectively operated as an acoustic reflector, allowing efficient acoustic transmission only through the apertures. The mask was coded using *P* = 41 and *Q* = 43, leading to a virtual array with *N* = 1763 elements and a theoretical SNR gain of ~21. The circular apertures had a diameter of 0.5 mm and were created on a grid with a spacing of Δ = 1 mm, leading to a coded detection area of 41 × 43 mm, and a full mask size of $\left(2P-1\right)\times \left(2Q-1\right)=6885$elements, corresponding to a mask area of $81\;\times\;85\;{{{{{\mathrm{mm}}}}}}$. Accordingly, at each scan position, only approximately a quarter of the mask surface overlaps with the detector. The use of a spacing that was twice the aperture size was essential for the mechanical stability of the mask but leads to a virtual array with gaps between the detectors. Nonetheless, since the aperture spacing was double their diameter, the mask may be translated with a step size of $\Delta /2=0.5\,{{{{{\rm{mm}}}}}}$, or a fraction thereof, to produce several virtual arrays with slight shifts between them, which may be combined into a single, gap-free array. The regions outside of the coded area were coated by an acoustically blocking porous polymer with 530 μm thickness (4701-30-25, PORON) to minimize parasitic signals that may be diffracted from the mask edges. A photograph of the coded mask is shown in Fig. 3A.

### System characterization measurement method

This section describes in detail the two performed measurements, characterizing the proposed detection setup. In the first measurement setup, the sensitivity of the virtual detection array was measured by comparison to a calibrated needle PVDF hydrophone (Precision Acoustics) with 1 mm diameter and 12 MHz bandwidth. A source transducer (A326s, Panametrics) was used to generate an acoustic wave with a central frequency of 5 MHz, which was initially measured by a hydrophone scanned in *x*-*y* plane, mapping the acoustic pressure field of the transducer. The measurement was repeated with the same source transducer and detected by the detector transducer (I8-0518-R, Harisonic) coded by the mask. The hydrophone from the first measurement and the masked detector from the second measurement was placed at the same distance of 12.7 cm from the source.

In the second measurement set, the resolution of the coded detector was measured by imaging an optoacoustic point source created at the tip of a gold-coated optical fiber. Optical pulses with 730 nm wavelength, pulse energy of 34 mJ, and pulse duration of 7 ns were coupled to the fiber from a free-space laser source (SpitLight EVO OPO, InnoLas), leading to optoacoustic generation of acoustic waves due to the optical absorption of the gold coating. The distance between the point source and the mask was 12.7 mm, and the distance between the mask and the single detector was 2.7 mm. The mask was scanned with a step size of 1 mm, equal to the grid spacing Δ, leading to a virtual detector array with 41 × 43 elements.

### Experimental hardware

The OAT setup, illustrated in Fig. 3B. included a pulse laser used to illuminate the imaged object (SpitLight EVO OPO, InnoLas), an acoustic mask, and a piezoelectric single-element unfocused transducer (I8-0518-R, Harisonic) with a 28.6 mm diameter, 5 MHz central frequency and 3 dB bandwidth of 3.1–6.5 MHz, which was placed behind the mask, at a distance of *d*, and used to detect the acoustic signals at the output of the mask. The laser produced wavelength-tunable pulses (680–980 nm) at a rate of 100 Hz, a duration of 7 ns, and typical energy of 30 mJ, which were delivered to the imaged specimen via a fiber bundle (CeramOptec). Two linear translation stages were used to scan the acoustic mask with discrete steps in the *x*-*y* plane (M-414.32 S and M-403.6PD, PI). The measured acoustic signal for each laser pulse corresponded to a single position of the acoustic mask. The diameter of the transducer was smaller than the size of the virtual array in order to avoid the detection of acoustic signals from apertures outside the virtual array, which would be interpreted as parasitic signals in the inversion of Eq. (4), as further explained in Supplementary Note 3 and shown in Supplementary Fig. 2. The voltage output of the transducer was passed through a 20 MHz low pass filter, amplified by 20 dB (DHPVA, Femto), and sampled by a data-acquisition unit with 16-bit voltage resolution (M3i.4860-Exp, Spectrum Instrumentation).

### Reporting summary

Further information on research design is available in the Nature Research Reporting Summary linked to this article.

### Supplementary information

Rosenthal_PR File

Supplementary Information

Description of Additional Supplementary Files

Supplementary Movie 1

Supplementary Movie 2

Reporting Summary

## Data Availability

Sampled ultrasound waveforms are available through zenodo: 10.5281/zenodo.6984211.

## Code availability

The written inversion code is available through zenodo: 10.5281/zenodo.6984211.
