# Supplementary material for: Single-detector 3D optoacoustic tomography via coded spatial acoustic modulation
Source: Commun Eng. 2022 Oct 19;1:25. doi: 10.1038/s44172-022-00030-7 (PMC10955898; doi:10.1038/s44172-022-00030-7)
Supplement: Supplementary file 2 — Supplementary Information [file 44172_2022_30_MOESM2_ESM.pdf]

Single-detector 3D optoacoustic tomography via coded spatial acoustic modulation

Supplementary material

Evgeny Hahamovich[1], Sagi Monin[1], Ahiad Levi[1], Yoav Hazan[1] and Amir Rosenthal[1]*

* Corresponding author. Email: amir.r@technion.ac.il
[1] Technion – Israel Institute of Technology

Supplementary note 1: Construction of the coding matrix

The coding matrix **W**, used to form the 2D patterns on the acoustic mask, was an S-matrix – a binary matrix that achieves the maximum SNR possible in the case of amplitude modulation[1]. While various construction methods for such a matrix exists, the need to code the matrix entries on a mask leads to the following requirements:

1. A cyclic structure to enable the scanning configuration of Fig. 2.
2. For a square matrix **W** with a size of N, it is required that N= PxQ to enable the mapping of the matrix elements to a mask with PxQ elements.
3. To enable detection surfaces that are close to square, it is required that $P \approx Q$.

Following those requirements, we created our optimal coding matrix based on the Twin-prime algorithm, that achieves optimal coding by using N of the form $N = PQ$ when P and Q are two following prime numbers and $Q = P + 2$ [1]. The Twin-prime algorithm for constructing such cyclic coding matrix is:

1. Choose a (P, Q) pair when both values are prime numbers and $Q = P + 2$.
2. Create a vector a with $N = P$xQ values in the range of $[0, (N - 1)]$.
3. Create the vector $c_f = \left[1,4, \dots \left((P-1)/2\right)^2\right]$.
4. Calculate the remainder from the division of a/P: $b_f = rem(a, P)$.
5. Calculate the remainder from the division of c_f/P: $d_f = rem(c_f, P)$.
6. Create a vector of N values equal to -1: $f(1:N) = -1$.
7. Add 1 to f in b_f indexes.
8. For i in the range of $[0, (Q - 1)]$: add 2 to the vector f in $d_f + iP$ positions.
9. Repeat steps 3-8 with Q and P replaced and save the output into vector f instead of g.
10. Create a vector with $(N - 1)$ values of 1: $v(1:n) = 1$.
11. Change values of v to 0 for indices satisfying at least one of the following:
    a. $f == g$.
    b. Indices $0, Q, 2Q, \dots (P - 1)Q$.
12. Assign to each mth row of the coding matrix **W** the vector v, cyclically shifted to the left by $(m - 1)$ values.

Our 2D mask was based on the pair of $(P, Q) = (41,43)$, leading to $N = 1763$ and a coding matrix **W** with 1763x1763 elements.

Supplementary note 2: 2D scanning grid formation

Assuming a cyclic code with a length of $N = P\text{x}Q$, formed using the procedure described in Sup. Note 1, the pattern of the acoustic mask is produced by mapping the 1D code elements onto the 2D surface. The pattern coded on the mask, described by the matrix **M**, has the dimensions of $(2P-1)\text{x}(2Q-1)$, where it is required that any contiguous subset of this pattern with dimensions $P\text{x}Q$, representing the detector integration area in Eq. 1, would yield a different cyclic shift of the 1D cyclic code, denoted by the vector v. This property is achieved by a recursive rule for the rows and column of **M:**

1. For each row in **M**, the element v_k is followed by the value of element $v_{(k+1)\bmod N}$.
2. For each column in **M**, the element v_k is followed by the value of element $v_{(k+Q)\bmod N}$.

In this configuration, a single-element shift of **M** in the horizontal direction is equivalent to a single-element cyclic shift of the vector v, whereas a single-element shift in the vertical direction is equivalent to cyclically shifting v by Q elements. Since the matrix **W** is composed of cyclic shifts of v, shifting the mask **M** is equivalent to choosing a different row of **W**.

An example for a mask **M** with coding size of $P\text{x}Q = 3\text{x}5$ and a convenient start from v_1 is shown in the following:

$$
\begin{matrix}
v_1 & v_2 & v_3 & v_4 & v_5 & v_6 & v_7 & v_8 & v_9 \\
v_6 & v_7 & v_8 & v_9 & v_{10} & v_{11} & v_{12} & v_{13} & v_{14} \\
v_{11} & v_{12} & v_{13} & v_{14} & v_{15} & v_1 & v_2 & v_3 & v_4 \\
v_1 & v_2 & v_3 & v_4 & v_5 & v_6 & v_7 & v_8 & v_9 \\
v_6 & v_7 & v_8 & v_9 & v_{10} & v_{11} & v_{12} & v_{13} & v_{14}
\end{matrix}
$$

Four coding positions over **M** are shown in Sup. Fig. 1. As can be seen, a vertical shift of the grid by a single grid step changes the coding line index in **W** from 1 to 2 in the translation from A to B and from 6 to 7 in the translation from C to D. In contrast, a horizontal shift of the grid by a single grid step shifts the coding line index by $Q = 5$, from 1 to 6 in the translation from A to C and from 2 to 7 in the translation from B to D.

A. $(q,p) = (1,1) \rightarrow v_1{:}v_{15} \rightarrow \mathbf{W}[1,:]$

v_1	v_2	v_3	v_4	v_5	v_6	v_7	v_8	v_9
v_6	v_7	v_8	v_9	v_{10}	v_{11}	v_{12}	v_{13}	v_{14}
v_{11}	v_{12}	v_{13}	v_{14}	v_{15}	v_1	v_2	v_3	v_4
v_1	v_2	v_3	v_4	v_5	v_6	v_7	v_8	v_9
v_6	v_7	v_8	v_9	v_{10}	v_{11}	v_{12}	v_{13}	v_{14}

B. $(q,p) = (1,2) \rightarrow v_2{:}v_1 \rightarrow \mathbf{W}[2,:]$

v_1	v_2	v_3	v_4	v_5	v_6	v_7	v_8	v_9
v_6	v_7	v_8	v_9	v_{10}	v_{11}	v_{12}	v_{13}	v_{14}
v_{11}	v_{12}	v_{13}	v_{14}	v_{15}	v_1	v_2	v_3	v_4
v_1	v_2	v_3	v_4	v_5	v_6	v_7	v_8	v_9
v_6	v_7	v_8	v_9	v_{10}	v_{11}	v_{12}	v_{13}	v_{14}

C. $(q,p) = (2,1) \rightarrow v_6{:}v_5 \rightarrow \mathbf{W}[6,:]$

v_1	v_2	v_3	v_4	v_5	v_6	v_7	v_8	v_9
v_6	v_7	v_8	v_9	v_{10}	v_{11}	v_{12}	v_{13}	v_{14}
v_{11}	v_{12}	v_{13}	v_{14}	v_{15}	v_1	v_2	v_3	v_4
v_1	v_2	v_3	v_4	v_5	v_6	v_7	v_8	v_9
v_6	v_7	v_8	v_9	v_{10}	v_{11}	v_{12}	v_{13}	v_{14}

D. $(q,p) = (2,2) \rightarrow v_7{:}v_6 \rightarrow \mathbf{W}[7,:]$

v_1	v_2	v_3	v_4	v_5	v_6	v_7	v_8	v_9
v_6	v_7	v_8	v_9	v_{10}	v_{11}	v_{12}	v_{13}	v_{14}
v_{11}	v_{12}	v_{13}	v_{14}	v_{15}	v_1	v_2	v_3	v_4
v_1	v_2	v_3	v_4	v_5	v_6	v_7	v_8	v_9
v_6	v_7	v_8	v_9	v_{10}	v_{11}	v_{12}	v_{13}	v_{14}

Supplementary Fig. 1. Example coding for $(\boldsymbol{P},\boldsymbol{Q}) = (\mathbf{3},\mathbf{5})$. Vector v has a total of $N = 15$ values. A-D show 4 different positions of the coding mask **M** over the detector area marked by the blue rectangular. A is the code from $\mathbf{W}[1,:]$, B is $\mathbf{W}[2,:]$, C is $\mathbf{W}[6,:]$, and D is $\mathbf{W}[7,:]$.

Supplementary note 3: Diffraction guided masking

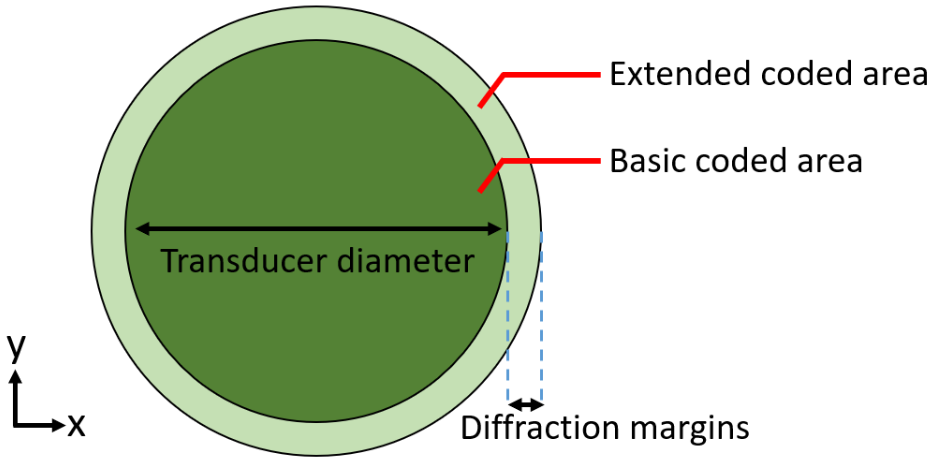

Supplementary Fig. 2. Diffraction impact on the coded area. The coded area in (x, y) plane includes the basic coded area, equal to the size of the transducer and an additional, extended coded area of the margins that are added to account for the diffraction of the signals during propagation from the coded mask to the detector.

The coding procedure described in Eq. 1 and Sup. Fig. 1 assumes that the detector integrates only over signals within the subset of PxQ elements, while all other signals are blocked completely. Ideally, for aperture spacing of Δ, a rectangular detector with an area of ΔPxΔQ would be used to cover all the PxQ elements on which the integration is to be performed. However, such a detector would also integrate signals from outside the PxQ subset due to diffraction: if the apertures are on the same scale as the acoustic wavelengths, their output will propagate semi-isotropically, and signals outside the desired detection surface may reach the detector diagonally. The result would be the addition of parasitic signals to the measured signals, which would lead to artifacts in the reconstruction.

Two measures were employed to minimize the detection of parasitic signals due to diffractions. First, the distance d between the detector and the mask was set to only a few millimeters, limiting the region from which the parasitic signals can emanate. Second, the detector dimensions were chosen to be smaller than ΔPxΔQ to assure that diffracted signals outside the detector area are still covered by the PxQ subset (Fig. S2). In our experimental setup, the detector had a round surface with a diameter of 29 mm, whereas the coded area was 41×43 mm, leaving a minimum of a 6 mm margins to each side of the detector. We note that the signals from the diffraction margins were still detected, and properly deconvolved, only with a lower efficiency that declined with distance from the detector surface. An additional advantage of the diffraction margins is that it reduced the effect of alignment errors between the mask and detector, as those mostly affected the attenuated signals from the margins.

We note that signals reaching the detector from the diffraction margins (Fig. S2) propagate a distance from the mask to the detector that is larger than d, which depends on the angle of incidence. Accordingly, it is expected that some temporal signal distortion would be obtained for those signals. Nonetheless, since only the apertures covered by the detector area (dark region in Fig. S2) are used in the reconstruction, this signal distortion does not affect the final outcome.

Supplementary note 4: Comparison with a random mask

We performed a numerical simulation comparing our scheme with an alternative scheme in which the mask is coded with a random binary pattern, rather than the S-sequence. The input image was composed of 20 spheres with varying diameters and amplitude, scattered over an area of 2×2 cm, with distances ranging from 2 mm to 10 mm from the surface of the acoustic mask. The mask had 201×205 aperture locations, where for each location, the transmission was either "1" or "0". In the first step, the acoustic signals were calculated on the surface of a virtual detector array with an area of approximately 2×2.04 cm divided over 101×103 elements, where each acoustic signal had a length of 685 points with a temporal resolution of 17 ns. In the second step, for each position of the mask, the acoustic signals were multiplied by the 101×103 elements of the mask in that position, and the result was integrated in space, leading to a single projected acoustic signal. Performing 101 and 103 scans in the horizontal and vertical directions led to a total of 101×103 projected acoustic signals. To each of the projected signals, white Gaussian noise was added with a zero mean and a standard deviation equal to 20 times the maximum value of the signals on the virtual array. This noise level corresponds to a realistic scenario in which the signals emanating from each of the apertures are too small to be individually detected in a direct non-multiplexed measurement. In the third step, the noisy projected signals were used to reconstruct the signal on the virtual detector array. For the mask coded with the S-sequence, the reconstruction was performed by merely multiplying by the inverse of the S-matrix, which is known analytically. In the case of the random mask, the reconstruction was performed using the iterative algorithm LSQR, which finds the signals that produce the projected data that is closest to the measured projected data under the L2 norm. The LSQR algorithm was performed with 30 iterations, where no significant improvement was noticed for a higher number of iterations. The reconstruction with the inverse S-matrix and LSQR required 1.2 s and 20 min, respectively, demonstrating the numerical advantage of using a closed-form inversion formula. In the last step, the signals from the virtual detector were used to reconstruct the input image.

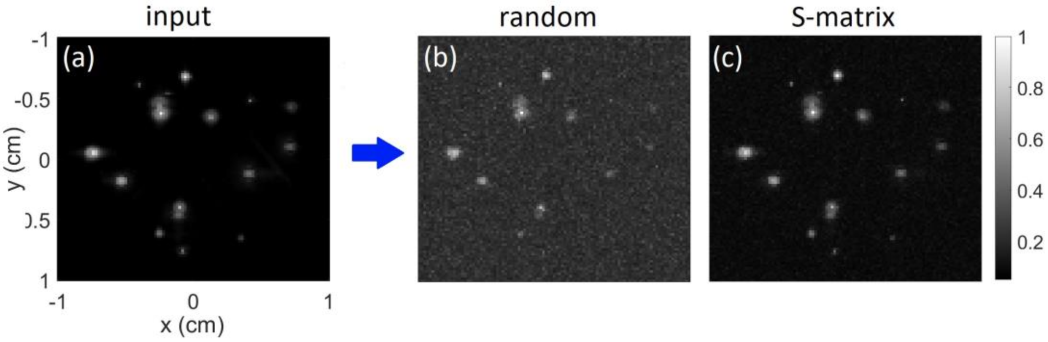

Supplementary Fig. 3. Comparison to a random mask. Maximum amplitude projections (MAPs) of (a) the input object, composed of spherical sources with varying diameters and depths (b,c) the numerical reconstructions obtained when the acoustic data was multiplexed with a random mask and mask coded with an S sequence, respectively. In both cases, the masks had 201×205 total apertures, where in each multiplexed measurement, the acoustic signals were multiplied by a mask region with the size of 101×103 and subsequentially spatially integrated. The reconstruction using a random mask led to 3-fold higher noise level, as can be visually appreciated from the images.

Supp Figs. 3a shows the maximum amplitude projection (MAP) of the noiseless reconstruction obtained from directly from the virtual signals calculated in Step 2, representing the ideal reconstruction scenario. Supp Figs. 3b and 3c respectively show the MAP of the noisy

reconstruction obtained from when using the random mask and the mask coded with the S-sequence. The figures clearly show that while both techniques succeeded in reconstructing the input image, the use of the S-sequence, which theoretically achieves the maximum SNR, indeed led to lower noise in the reconstruction. Specifically, the root mean square error in the reconstructions of Figs. 3b and 3c, in comparison to the noiseless reconstruction of Fig. 3a, were 0.075 and 0.21, respectively, representing an approximately 3-fold SNR advantage for the S-sequence mask.

Supplementary note 5: Robustness against the position of the mask

While our theoretical model assumes that the acoustic signals are integrated on the surface of the mask, the experimental setup includes an additional propagation distance, d, due to geometrical constraints of the system (Fig. 3b). We experimentally tested the effect of d on the optoacoustic reconstruction for an object composed of four pencil leads, made of graphite, embedded in a clear agar phantom. The coded mask used in these measurements was the same one used in the main text. The object was imaged three time, where for each time a different value of d was used: 1 mm, 3 mm, and 5 mm. The MAP of the three reconstructions is shown in Sup. Fig. 4, revealing no significant difference between the reconstructions.

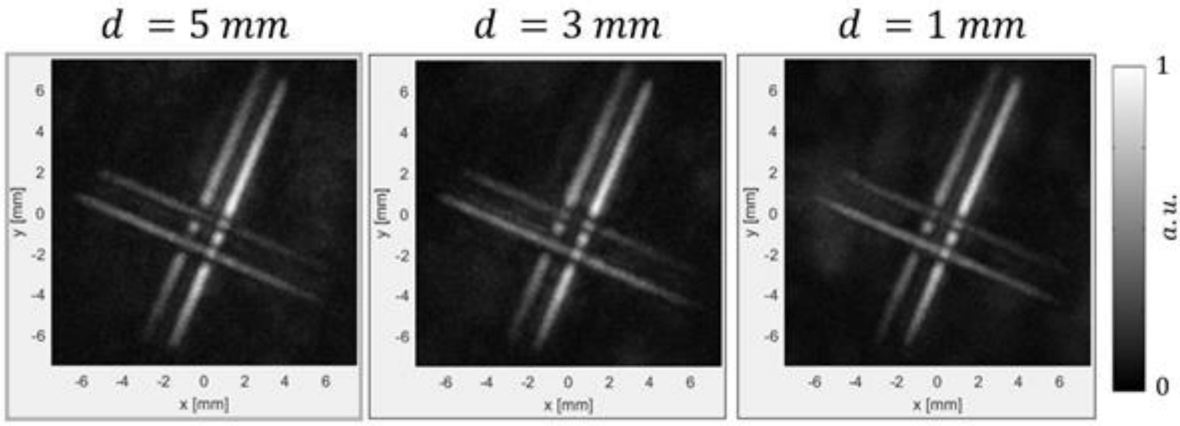

Supplementary Fig. 4. The effect of the mask distance from the detector, d. A phantom composed of 4 pencil leads, made of graphite, was imaged for 3 values of d: 1, 3, and 5 mm. The figure shows no significant difference between the reconstructions, demonstrating that the exact value of *d* is not a crucial parameter in these ranges in our setup.

Supplementary References

1. Harwit, M. *Hadamard transform optics*. (Elsevier, 1979). doi:10.1016/B978-0-12-330050-8.50001-9
