# Supplementary material for: Single-detector 3D optoacoustic tomography via coded spatial acoustic modulation
Source: Commun Eng. 2022 Oct 19;1:25. doi: 10.1038/s44172-022-00030-7 (PMC10955898; doi:10.1038/s44172-022-00030-7)
Supplement: Supplementary file 3 — Description of Additional Supplementary Files [file 44172_2022_30_MOESM3_ESM.docx]

Description of Additional Supplementary Files

**File name:** Supplementary Movie 1

**Description: OAT reconstruction of a** **complex suture.** The suture OAT reconstruction is created from a set of acoustic signals, decoded from a scrambled captured dataset. This animation corresponds to Fig. 5.D in the main manuscript, with x and y axes in millimeters and the grayscale colorbar representing the optical absorption in arbitrary units (a.u.).

**File name:** Supplementary Movie 2

**Description: OAT reconstruction of a mouse leg in-vivo.** The 3D OAT reconstruction is created from a set of acoustic signals, decoded from a scrambled captured dataset. This animation corresponds to Fig. 6.D in the main manuscript, with x and y axes in millimeters and the grayscale colorbar representing the optical absorption in arbitrary units (a.u.).
