# Supplementary material for: Single-detector 3D optoacoustic tomography via coded spatial acoustic modulation
Source: Commun Eng. 2022 Oct 19;1:25. doi: 10.1038/s44172-022-00030-7 (PMC10955898; doi:10.1038/s44172-022-00030-7)
Supplement: Supplementary file 1 — Rosenthal_PR File [file 44172_2022_30_MOESM1_ESM.pdf]

nature portfolio

Peer Review File

SSingle-detector 3D optoacoustic tomography via coded spatial acoustic modulation

Open Access This file is licensed under a Creative Commons Attribution 4.0 International License, which permits use, sharing, adaptation, distribution and reproduction in any medium or format, as long as you give appropriate credit to the original author(s) and the source, provide a link to the Creative Commons license, and indicate if changes were made. In the cases where the authors are anonymous, such as is the case for the reports of anonymous peer reviewers, author attribution should be to 'Anonymous Referee' followed by a clear attribution to the source work. The images or other third party material in this file are included in the article's Creative Commons license, unless indicated otherwise in a credit line to the material. If material is not included in the article's Creative Commons license and your intended use is not permitted by statutory regulation or exceeds the permitted use, you will need to obtain permission directly from the copyright holder. To view a copy of this license, visit http://creativecommons.org/licenses/by/4.0/.

Reviewers' comments:

Reviewer #1 (Remarks to the Author):

The manuscript develops a novel scheme for single-detector OAT based on the amplitude mask and the planar configuration. It is possible to obtain 3D OA images with linear operations of reconstruction, which can obtain high SNR and is compatible with spectral analysis. The authors may consider the following comments.

In Eq. 5, where d is the distance between the mask and the single detector.
-- Is d the vertical distance from mask to transducer? If so, is this parameter as small as possible?
-- The diameter of the transducer used in this manuscript is 28.6 mm (Fig.S1, red). For holes far from the aperture of the transducer (Fig.S2, white with '? '), and how to calculate the value of this parameter d?
-- Different d values are used in the actual experiment, especially in the experiment of complex samples, the d value is larger. Is it necessary to set the optimal distance for different PA source in this method? Please explain the reasons.
Since mask (Fig.S2, black and white) is 41*43, and virtual detection (Fig.S2, green) is also 41*43 in this manuscript, will the mask be swept out at the edge? Does this situation lead to error? Please explain the reasons.

In the actual experiment, I think the most critical experimental step is the registration of the mask pattern at each scanning position and matrix W (especially the mask at the first scanning position and the first row of matrix W should be completely corresponding). Could you expand it in detail and explain how to do this? In addition, in the scanning process, the scanning speed of 10 mm/s is adopted. Due to the acceleration and deceleration of the translation platform, the registration difficulty is increased. How do you deal with it?

The figures should be added with the colorbar, such as Fig4, Fig5, Fig6. In addition, it has been mentioned many times that the theoretical SNR is enhanced by 21-fold, so can the actual SNR value be supplemented in the imaging results?

Reviewer #2 (Remarks to the Author):

The manuscript describes a promising design for the development of high-resolution, low-cost optoacoustic tomography (OAT) systems. It uses a high-frequency laser, a single ultrasound detector, and a coded spatial acoustic modulation mask. By carefully engineering the coding of the mask and by horizontally and vertically scanning the mask, a virtual 2D transducer array is obtained.

Main strengths:

- The proposed design has the promise to allow for a new generation of high-fidelity low-cost OAT systems, using a single detector and relatively inexpensive electronics.
- The proposed data acquisition paradigm allows for use of standard well-established OAT reconstruction algorithms (such as Universal Backprojection Algorithm) with minimal modifications.
- The overall image quality is superior to that obtained by systems using a planar detection surface with a 2D transducer array.

Main weaknesses:
- In the manuscript, it is not clearly stated which imaging applications (e.g. small animal imaging) are the main target of the proposed system
- In the discussion, it is reported that the achieved resolution is 150μm in the axial direction and

480μm in the lateral direction. However, no experimental/phantom data are shown (either in the manuscript or supporting document) to support the statement. Also, a resolution of 480μm may be too coarse to support any meaningful small animal imaging applications
- The in-vivo results are not convincing. Several small-animal OAT systems are able to clearly show vasculature structures within the animal. In the manuscript, the authors purposely chose a wavelength of ~530nm for the in vivo experiment (so that deep tissue can not be imaged due to the strong absorption by the skin layer) when lasers in the 700-800nm range were also available (and that would have indeed allowed for deep tissue imaging)

Suggested revisions:
- Clearly explain (either in the introduction or discussion) which imaging applications can be targeted using the proposed system design.
- Provide more experimental evidence (such as full width at half maximum analysis of a simple "resolution" phantom consisting of tubes of different diameters oriented along with different directions) or a reference to where these studies were performed.
- Provide a more compelling in-vivo imaging study

Reviewer #3 (Remarks to the Author):

The authors report a method for structured spatial acoustic modulation using an amplitude mask coded with cyclic patterns for optoacoustic tomography. The proposed method is compatible with planar signal detection, and enable image reconstruction using linear operations. The experiments show that the method is capable of 3D OAT of complex objects and living tissue. The general idea of using a mask in front of a single ultrasound transducer for 3D ultrasound imaging is not new, as also pointed out by the authors. The main novelty of the current paper is to use structured (repeated) patterns rather than random codes on the mask. In general, the theory and simulation well explain the phenomenon and the paper is clearly written. The reviewer has questions and minor comments as below.
1. What is the biggest advantage of the structured mask comparing to the random masks that were already reported? Experimental proof or simulation results should be provided to support the comparison.
2. What is the criteria for the optimization of the codes on the mask? The authors mentioned briefly the optimization method in the SI, however, it is not entirely clear how it was done. And no experimental evidence to show the current is indeed optimized.
3. The authors mentioned that the method increases the angular acceptance of the detector. A detailed explanation with data support would be needed.
4. Would an amplitude-modulation mask be better than the current binary mask? For example, to obtain more information in each scan and thus increase the scanning speed?

Reviewers' comments:

Reviewer #1 (Remarks to the Author):

The manuscript develops a novel scheme for single-detector OAT based on the amplitude mask and the planar configuration. It is possible to obtain 3D OA images with linear operations of reconstruction, which can obtain high SNR and is compatible with spectral analysis. The authors may consider the following comments.

Reply: We thank the Reviewer for the detailed and thorough feedback, which helped us improve the quality of our manuscript. In the following is a point-by-point reply to the Reviewer's comments. Modifications in the text are highlighted in a separate version.

In Eq. 5, where d is the distance between the mask and the single detector.
-- Is d the vertical distance from mask to transducer? If so, is this parameter as small as possible?
-- The diameter of the transducer used in this manuscript is 28.6 mm (Fig.S1, red). For holes far from the aperture of the transducer (Fig.S2, white with '? '), and how to calculate the value of this parameter d?
-- Different d values are used in the actual experiment, especially in the experiment of complex samples, the d value is larger. Is it necessary to set the optimal distance for different PA source in this method? Please explain the reasons.

Reply: As now explained in the manuscript, d is the distance between the planes of the mask and the detector. It is a single value, and not the distance between individual holes to the detector. We have updated Fig. 3b to include d. Accordingly, all the apertures covered by the area of the detector have the same value of $d.$ Apertures outside the detector coverage (the diffraction margins in Fig. S2) effectively have a slightly larger d due to the angle, which would lead to distortion in the reconstruction of the signals in these regions. However, the regions outside the mask coverage are not used in the optoacoustic reconstruction, but rather discarded, and the effect of signal distortion in these regions does not affect the optoacoustic reconstruction. This issue is now addressed in Supp. Note 2.

In principle, if we assume that the detector performs integration over the wave at the output of the mask, "d" needs to be ideally zero, or more practically smaller than the acoustic wavelength (or in the near field of the apertures). In practice, however, considerably larger distances may be used, as now experimentally demonstrated in Supplementary Note 5. Specifically, we performed 3 imaging measurements of a phantom made from 4 pencil leads (made from graphite), where for each measurement, a different value of "d" was used: 1 mm, 3 mm, 5 mm. As can be seen from the figure, the same reconstructions are obtained in all three examples. Since the exact value of "d" is not crucial, the different values of "d" used in the other measurements were chosen mainly due to experimental constraints.

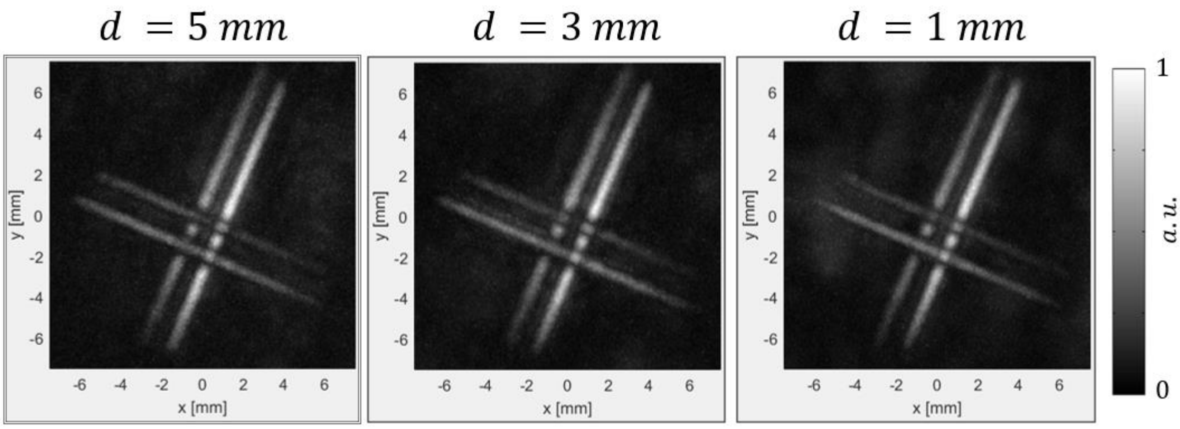

The ability to position the detector at a distance from the mask without significant signal deformation may be explained by the response of flat acoustic detectors, and we added an explanation in the Discussion. When the spherical wavefront of the acoustic wave impinges on the flat detector, the first point of contact, where the wavefront is tangential to the detector surface (and the mask) dominates the response, whereas of the rest of the wavefront, which is a result of acoustic diffraction, is effectively averaged out. In our setup, the first point of contact occurs at a delay of "d/c" and therefore one may approximate the propagation from the aperture to the detector as merely a propagation of a distance "d", neglecting all the contribution of the spherical wave that are not in the z direction (Fig. 3). Nonetheless, the distance between the mask and detector should still be kept small in comparison to the detector/mask size to reduce the contribution of signals from outside the detector area due to diffraction (Fig. 2 in the Supplementary Materials).

Since mask (Fig.S2, black and white) is 41*43, and virtual detection (Fig.S2, green) is also 41*43 in this manuscript, will the mask be swept out at the edge? Does this situation lead to error? Please explain the reasons.

As now explicitly stated in the revised version of the manuscript, the mask area is approximately 4 times larger (in area) than that of the detector, and only a quarter of its elements are used for coding in each mask position. This geometry is also illustrated in Sup. Fig.1, where the mask is 9x5 elements but the detector is only 5x3 elements. More generally, the coded area is a rectangular of size $P\text{x}Q$ and this is also the size of the formed virtual detector. The coding mask is larger, with $(2P-1)\text{x}(2Q-1)$ dimension, allowing it to be scanned over P vertical positions and Q horizontal positions sweeping over the edge. In the example of sup. Fig. 1 $PxQ = 3x5$ and the full coded mask area is $(2P-1)\text{x}(2Q-1) = 5\text{x}9$ and in the real experiment $PxQ = 41\text{x}43$ and the full coded mask area is $(2P-1)\text{x}(2Q-1) = 81\text{x}85$.

In the actual experiment, I think the most critical experimental step is the registration of the mask pattern at each scanning position and matrix W (especially the mask at the first scanning position and the first row of matrix W should be completely corresponding). Could you expand it in detail and explain how to do this? In addition, in the scanning process, the scanning speed of 10 mm/s is adopted. Due to the acceleration and deceleration of the translation platform, the registration

difficulty is increased. How do you deal with it?

Indeed, the exact knowledge of each mask position is crucial and the sampling system is synchronized with the translation stages. As now explained in the text, to avoid non-constant scanning speed during the signal acquisition, we added acceleration and stopping paths to the beginning and end of the motion. Accordingly, the mask was scanned a bit extra in the continuous axis, where the extra measurements were discarded.

The figures should be added with the colorbar, such as Fig4, Fig5, Fig6. In addition, it has been mentioned many times that the theoretical SNR is enhanced by 21-fold, so can the actual SNR value be supplemented in the imaging results?

We have added a color bar to Figs. 5 and 6, whereas in Fig. 4 1D slices were added to visualize the change in amplitude across the reconstruction. Since the images are presented in arbitrary units, the color bar does not have numerical values. In addition, as mentioned in the “System Characterization Measurements”, the sensitivity of the virtual detection elements was characterized, and it was found they have a noise equivalent pressure (NEP) of 200 Pa. This measured value is the true representation of the performance of the detector elements, which includes the 21-fold sensitivity increase. In contrast, the SNR in the images does not represent an intrinsic property of the system, and is heavily affected by the illumination configuration, and thus a separate SNR analysis for each dataset does not contribute to the understanding of the performance of our technique.

Reviewer #2 (Remarks to the Author):

The manuscript describes a promising design for the development of high-resolution, low-cost optoacoustic tomography (OAT) systems. It uses a high-frequency laser, a single ultrasound detector, and a coded spatial acoustic modulation mask. By carefully engineering the coding of the mask and by horizontally and vertically scanning the mask, a virtual 2D transducer array is obtained.

Reply: We thank the Reviewer for the detailed and thorough feedback, which helped us improve the quality of our manuscript. In the following is a point-by-point reply to the Reviewer's comments. Modifications in the text are highlighted in a separate version.

Main strengths:

- The proposed design has the promise to allow for a new generation of high-fidelity low-cost OAT systems, using a single detector and relatively inexpensive electronics.
- The proposed data acquisition paradigm allows for use of standard well-established OAT reconstruction algorithms (such as Universal Backprojection Algorithm) with minimal modifications.
- The overall image quality is superior to that obtained by systems using a planar detection surface with a 2D transducer array.

Main weaknesses:
- In the manuscript, it is not clearly stated which imaging applications (e.g. small animal imaging) are the main target of the proposed system
- In the discussion, it is reported that the achieved resolution is 150µm in the axial direction and 480µm in the lateral direction. However, no experimental/phantom data are shown (either in the manuscript or supporting document) to support the statement. Also, a resolution of 480µm may be too coarse to support any meaningful small animal imaging applications
- The in-vivo results are not convincing. Several small-animal OAT systems are able to clearly show vasculature structures within the animal. In the manuscript, the authors purposely chose a wavelength of ~530nm for the in vivo experiment (so that deep tissue can not be imaged due to the strong absorption by the skin layer) when lasers in the 700-800nm range were also available (and that would have indeed allowed for deep tissue imaging)

Suggested revisions:
- Clearly explain (either in the introduction or discussion) which imaging applications can be targeted using the proposed system design.

We have added explanation on imaging applications that may benefit from our new approach in the Discussion of the revised version of the manuscript. Since the system relies on mechanical scanning, we focus on systems performed with mechanical scanning today. One application is whole-body small animal imaging, e.g., using multi-spectral optoacoustic tomography (MSOT), which is commonly performed with curved ultrasound transducers. Using a flat geometry could simplify the animal handling and the use of a single detector would significantly reduce the cost. An alternative application is optoacoustic microscopy which is currently being performed with a focused detector that is raster-scanned above the imaged region. The advantage of using a tomographic scheme is that the imaging depth will not be limited to the depth of field of the transducer.

- Provide more experimental evidence (such as full width at half maximum analysis of a simple "resolution" phantom consisting of tubes of different diameters oriented along with different directions) or a reference to where these studies were performed.

The spatial resolution of our system was determined by the width of its point-spread function (PSF), measured in Fig. 4. While the original version of the manuscript only showed the maximum amplitude projection (MAP) of the three-dimensional PSF, the current version shows 3 slices along its three principal axes. The calculation of the full width at half maximum from these slices is now also shown in the figure. Fig. 4 also shows that the PSF has a short oscillation in the z direction, which included negative values. This oscillation is theoretically expected from the lack of low frequencies in the response of the transducer. To estimate the PSF width in that direction, we first calculated the envelope of the reconstruction, which led to a width of 320 µm. This width is in agreement with the theoretical prediction of 0.88 c/BW $\approx 380\,\mu$m [Lihong V. Wang and Song Hu, Science, 2012, DOI: 10.1126/science.1216210], where c is the speed of sound in water, approximately equal to 1500 m/s and BW is the detector bandwidth, approximately equal to 3.4 MHz in our setup. We note that in the original version of the manuscript, only the positive part of the PSF, rather than its envelope was used to estimate the axial width, leading to the wrong estimate of 150 µm. We thank the Reviewer for asking for a more detailed analysis, which led us to find this miscalculation.

- Provide a more compelling in-vivo imaging study

As noted by the Reviewer, the current implementation of our technique with a lateral resolution of 480 µm limited our ability to perform *in vivo* imaging of small animals. This resolution is a result of the aperture sizes used in the mask and the central frequency of the transducer, and not an inherent limitation of the technique. Additionally, our imaging setup was designed for transmission mode, which prevented imaging of deep regions. Indeed, most deep-tissue optoacoustic systems operate in reflection mode, which enables an efficient delivery of light to the tissue. As the Reviewer can probably appreciate, performing *in vivo* studies with a scanning OAT system involves numerous technical challenges (e.g. efficient light delivery to the tissue, motion artifacts, submersion of the animal, etc.). Unfortunately, our current capabilities with *in vivo* OAT do not fully match the technical specifications of the system and its current limitations. However, the goal of our work was not to develop a specific system for a given imaging application, but rather demonstrate a new imaging concept based on acoustic apertures. As discussed in the Discussion, the system may be adapted to reflection mode by using recently developed transparent ultrasound transducers and transparent masks (e.g. from glass) to enable imaging of deeper targets. We hope that the Reviewer can appreciate that the newly added PSF (Fig. 4), which is in full agreement with optoacoustic theory, and the 3D image of a complex phantom (Fig. 5) provide sufficient proof that the use of acoustic masks enables high-quality OAT in planar geometries, even if the current implementation has not been optimized for *in vivo* studies.

Reviewer #3 (Remarks to the Author):

The authors report a method for structured spatial acoustic modulation using an amplitude mask coded with cyclic patterns for optoacoustic tomography. The proposed method is compatible with planar signal detection, and enable image reconstruction using linear operations. The experiments show that the method is capable of 3D OAT of complex objects and living tissue. The general idea of using a mask in front of a single ultrasound transducer for 3D ultrasound imaging is not new, as also pointed out by the authors. The main novelty of the current paper is to use structured (repeated) patterns rather than random codes on the mask. In general, the theory and simulation well explain the phenomenon and the paper is clearly written. The reviewer has questions and minor comments as below.

Reply: We thank the Reviewer for the detailed and thorough feedback, which helped us improve the quality of our manuscript. In the following is a point-by-point reply to the Reviewer's comments. Modifications in the text are highlighted in a separate version.

Regarding the novelty of our work, we would like to emphasize that in addition to the use of structured (rather than random) patterns, our work includes the additional novelty of using wavelength-scale apertures to enable semi-isotropic resolution OAT in a planar geometry. Previous works in which random acoustic structures were used did not demonstrate this important property, which is not a result of the specific pattern used, but rather of the very specific diffraction properties of acoustic apertures.

1. What is the biggest advantage of the structured mask comparing to the random masks that were already reported? Experimental proof or simulation results should be provided to support the comparison.

We added a simulation study in Supp. Note 4 in which our coded mask was compared with a random mask, demonstrating two advantages: higher SNR and faster reconstruction times. Based on multiplexing theory (Ref. [39]), the binary codes used in our work (S-sequence) achieve the upper bound for SNR for any amplitude mask. Indeed, when compared to mask with a random code in the new simulation study, the reconstruction SNR was 3 times higher. In terms of reconstruction efficiency, the advantage of using an S-matrix for multiplexing is that its inverse is know analytically. Thus, the reconstruction involves merely multiplying the known inverse matrix by the data. In contrast, image formation with a random matrix is generally performed in an iterative procedure, where in our type of problem, the inversion needs to be separately performed to each time point (685 time points in total). In our iterative implementation with LSQR, the reconstruction via a random matrix required 20 minutes, whereas only 1.2 seconds were required for inversion with the inverse S matrix.

2. What is the criteria for the optimization of the codes on the mask? The authors mentioned briefly the optimization method in the SI, however, it is not entirely clear how it was done. And no experimental evidence to show the current is indeed optimized.

Our mask is based on well-established cyclic codes, which have been mathematically proven to asymptotically achieve the maximum SNR under the assumption of additive non-correlated noise (Ref. [39]). Accordingly, we did not optimize the codes ourselves, but rather used a known algorithm to generate them, described in Supp. Note 1. This property of optimality, in comparison with other amplitude masks, is now further discussed in the Discussion.

3. The authors mentioned that the method increases the angular acceptance of the detector. A detailed explanation with data support would be needed.

We have conducted a thorough study on the increase in angular acceptance angle due to the use of apertures in our previous work (Refs. [36] and [37]), and was explained by acoustic diffraction. There we showed acceptance angles up to ±50 degrees, whereas conventional piezoelectric transducers are generally limited to ±10 degrees. The exact value of the acceptance angle depends on the specific details of the implementation, mainly the width and depth of the apertures.

The effect of a low acceptance angle is deterioration in lateral resolutions, leading to highly anisotropic images with up to 1:10 relation between the axial and lateral resolutions in flat geometries based on piezo-electric transducers ([Vionnet, L. et al. IEEE Trans. Med. Imag. 33, 535–545 (2014).]). The axial are lateral resolutions achieved in our work were 480 and 320 µm, respectively, i.e., a relation of 1:1.5. This semi-isotropic resolution in a planar geometry is a clear indication of the large acceptance angle and is exactly the reason why a large acceptance angle is desired.

4. Would an amplitude-modulation mask be better than the current binary mask? For example, to obtain more information in each scan and thus increase the scanning speed?

Multiplexing theory states that any positive amplitude multiplexing pattern can achieve at most a $\sqrt{N}/2$ SNR gain with respect to a non-multiplexed measurement. Our cyclic binary codes approximately reach this limit and therefore there is no advantage of using other amplitude codes that are not binary. In contrast, if negative values are allowed, which can be achieve by a phase mask, then an SNR gain of $\sqrt{N}$ is possible. However, addition a phase of π to a given element in the mask, e.g. by using a material with a different speed of sound, can be performed only at a specific acoustic wavelength. Thus, producing "-1" values with phase masks would limit the bandwidth of the system. In any case, regardless of the code used, exact demultiplexing of N pixels (i.e. without using nonlinear inversion algorithms of compressed sensing) would require N measurements, regardless of the codes are binary or not.

REVIEWERS' COMMENTS:

Reviewer #1 (Remarks to the Author):

The manuscript develops a novel scheme for single-detector OAT based on the amplitude mask and the planar configuration. It is possible to obtain 3D OA images with linear operations of reconstruction, which can obtain high SNR and is compatible with spectral analysis.The results (resolution and depth, etc.) of the in vivo experiments carried out in this paper are not ideal, but the method itself is novel and has reference value, which can provide more thinking for low-cost photoacoustic imaging.
I suggest that the article be accepted

Reviewer #2 (Remarks to the Author):

I would like to thank the authors for the careful and detailed manuscript revision. The additional experimental and numerical studies performed by the authors are excellent and provide further evidence of the potential impact of the proposed method.

I have no further concerns and I would like to recommend the revised manuscript for publication in its present form.

NOTE: In the supplementary material the figures numbering skips from 2 to 4. I recommend fixing this before final submission.

Reviewer #3 (Remarks to the Author):

The authors have revised the manuscript following the comments from all three reviewers, and the quality of the paper has been further improved. I do not have further questions, and would recommend the publication of the paper.
